# Realtime phase-amplitude coupling analysis of micro electrode recorded brain signals

**DOI:** 10.1371/journal.pone.0204260

**Published:** 2018-09-28

**Authors:** David Chao-Chia Lu, Chadwick Boulay, Adrian D. C. Chan, Adam J. Sachs

**Affiliations:** 1 Department of Systems and Computer Engineering, Carleton University, Ottawa, Ontario, Canada; 2 Department of Neurosciences, The Ottawa Hospital Research Institute, Ottawa, Ontario, Canada; 3 Faculty of Medicine and Brian and Mind Research Institute, The University of Ottawa, Ottawa, Ontario, Canada; Mar Ephraem College of Engineering & Technology, INDIA

## Abstract

**Objective:**

To demonstrate a method to calculate phase amplitude coupling (PAC) quickly and robustly for realtime applications.

**Methods:**

We designed and implemented a multirate PAC algorithm with efficient filter bank processing and efficient computation of PAC for many frequency-pair combinations. We tested the developed algorithm for computing PAC on simulated data and on intraoperative neural recording data obtained during deep brain stimulation (DBS) electrode implantation surgery.

**Results:**

A combination of parallelized frequency-domain filtering and modulation index for PAC estimation provided robust results that could be calculated in real time on modest computing hardware.

**Conclusion:**

The standard methods for calculating PAC can be optimized for quick and robust performance.

**Significance:**

These results demonstrated that PAC can be extracted in real time and is suitable for neurofeedback applications.

## Introduction

Deep brain stimulation (DBS) of the subthalamic nucleus (STN) is a common treatment for advanced motor-predominant Parkinson’s disease (PD) [1–3]. The exact mechanism of DBS remains unclear, but STN stimulation has shown to improve the main motor symptoms of PD [4, 5]. DBS efficacy and patient tolerance to stimulation depend greatly on accurate and precise electrode placement [6], which relies on preoperative surgical planning and intraoperative neurophysiological mapping.

Neurophysiological mapping requires a site-by-site inspection of microelectrode recorded (MER) signals by experts, and is time consuming and laborious. This process can be automated and optimized utilizing state of the art digital signal processing and machine learning techniques along with the knowledge of Parkinsonian STN. For example, abnormal beta band (*β*: 14-30 Hz) oscillations were observed in local field potentials via DBS electrodes of PD patients suffering from bradykinesia and rigidity [7]. Recent evidence suggested that Parkinsonian motor dysfunctions may not be due to abnormal *β* power alone, but rather its effect on the entire spectra of neural activity, including broadband gamma (*γ*_2_: 50-200 Hz) and higher frequency oscillations (HFO: 200-500 Hz) [4].

Phase-amplitude coupling (PAC) is used to analyze the interactions between frequencies within a signal [4, 8–11]. PAC magnitude is proportional to the level of synchronization between the phase of the lower frequency rhythm signal and the amplitude envelope of the higher frequency rhythm signal.

PAC is most commonly used in studies of rhythmic relationships of brain signals, and may represent a method for communication within and between distinct anatomical regions of the brain [12, 13]. Studies have shown that *β*–*γ*_2_[12] and *β*–HFO [4, 14] PAC are present in the basal ganglia of PD patients, and PAC reduction during therapy is observed with symptom improvement [13]. Currently, realtime estimation of *β* power is being investigated as a signal to operantly condition to reduce PD dysfunction [15] or as a trigger for DBS stimulation [16, 17]. As *β*–*γ*_2_ or *β*–HFO PAC may be a better biomarker of dysfunction in PD than *β* power alone [4, 12, 13], realtime PAC estimation may be valuable in these applications as well.

There are a number of described methods to calculate PAC [9, 10]. In most of these methods, the first step is to obtain the analytic signal for each of the low- and high-frequency components of the source signal. The second step is to quantify PAC, either by mapping the amplitude-phase information to polar coordinates, by using the phase-amplitude distribution [4, 8–11], or by statistical determination of the correlation/coherence between the phase signal and the amplitude envelopes. These steps are computationally intensive and cannot support real time use with current processing limitations. Further, it is often necessary to calculate PAC separately for many combinations of low- and high-frequency band pairs because the coupling frequencies vary among individuals and cannot be predicted precisely. Computation time increases exponentially with each frequency added to the test set.

PAC is traditionally used in offline analysis only, where computation time is not a major constraint and signal artefacts can be mitigated prior to PAC processing. However, for PAC to be useful in realtime applications, such as to assist with DBS electrode localization [1–3, 18] or closed loop stimulation control in the treatment of PD [16, 17], PAC must be calculated quickly and robustly [19]. In this study, we investigate different PAC calculation methods and their suitability for realtime applications. We developed a customized approach to calculate PAC and optimized its parameters for analysis of human intracranial recordings obtained during DBS implantation surgery.

## Materials and methods

### Phase-amplitude coupling (PAC)

**Fig 1:**
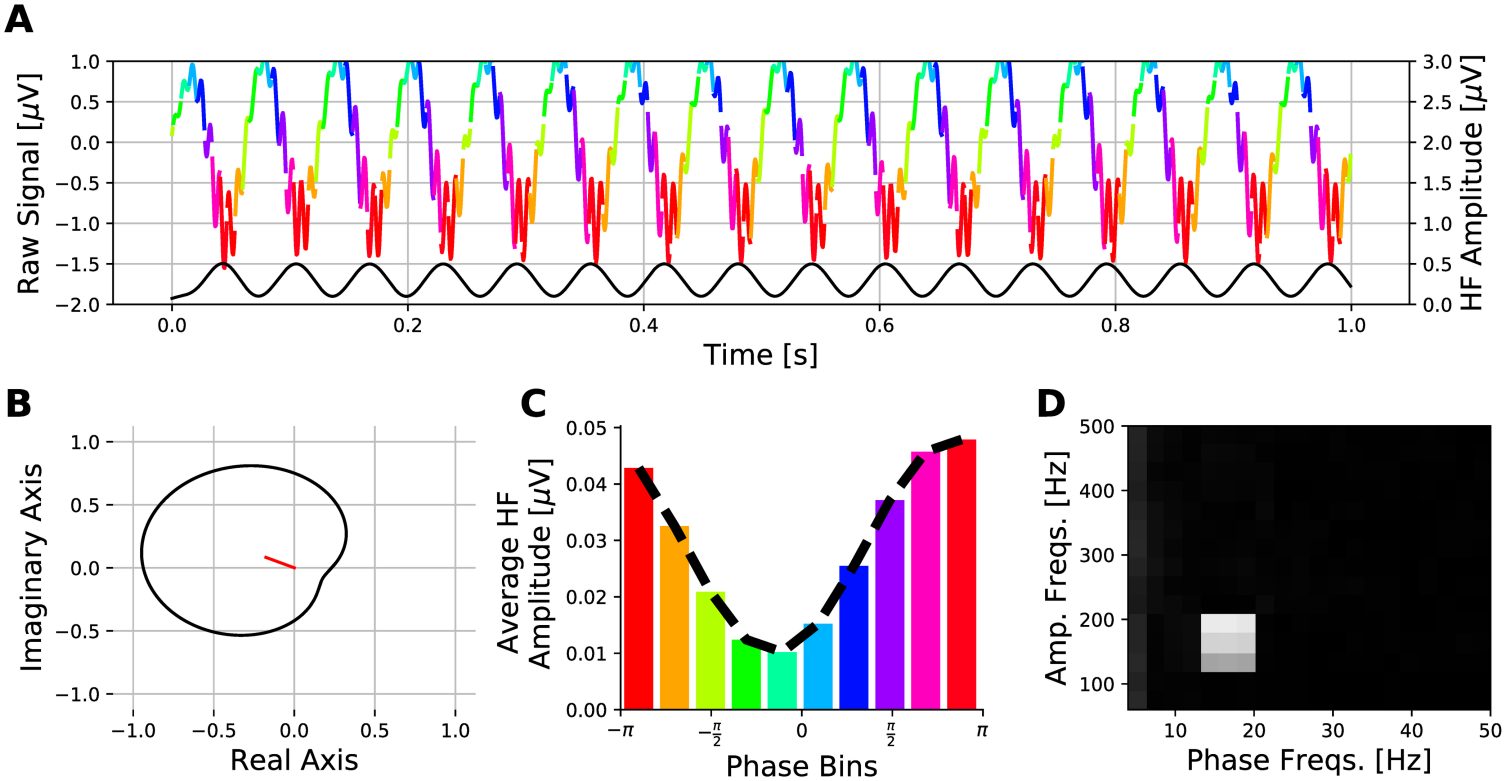
An illustrative description of PAC. A: The top multi-colored line depicts a generated signal containing PAC. Each line segment’s colour indicates the phase bin of the 16 Hz signal corresponding to the binning scheme in panel C. The bottom line is *A*(*n*), the norm of *x*_*a*_(*n*). B: A polar plot, defined with Eq (1), of *A*(*n*) as a function of *ϕ*(*n*). The mean vector is shown in red and mean vector length (MVL) is one quantification of PAC magnitude. C: The phase-amplitude distribution is constructed by binning A(n) according to the corresponding values in *ϕ*(*n*) and averaging within each bin. This test signal has higher amplitudes on its way to the trough of *xp*(*n*), and lower amplitudes on its way to the peak. D: PAC magnitude for many different combinations of frequency pairs is presented in a comodulogram. There are 20 different centre low frequencies between 4-50 Hz used for phase. There are 15 different centre high frequencies between 60-250 Hz used for amplitude. The colour indicates the PAC magnitude (using MI) for each frequency pair.

Many algorithms for quantifying PAC have been described [8–10, 20–22], each with relative strengths for different applications. Fig 1 illustrates a signal containing PAC and some visualizations of PAC quantification. The signal is filtered to obtain its low frequency (14-30 Hz) and high-frequency (80-200 Hz) oscillations. The Hilbert transform is applied to each filtered signal to express them in analytical form, then the instantaneous phase of the low-frequency signal (*ϕ*(*n*)) and the instantaneous amplitude of the high-frequency signal (*A*(*n*)) are obtained. These phase and amplitude signals are then used to quantify PAC.

#### PAC quantifications

Some methods for quantifying PAC in a signal map *A*(*n*) and *ϕ*(*n*) to the polar form, as illustrated in Fig 1B. This can be mathematically expressed as

$$\begin{array}{c} z\left(n\right)=A\left(n\right)e^{j\phi (n)}, \end{array}$$ (1)

where *n* is the index sample. In the absence of PAC, *A*(*n*) is a step function and *z*(*n*) is a circle. This circle deforms with PAC emerging in the signal.

The mean vector length (MVL) is used for quantifying PAC from the polar form. The MVL is defined as

$$\begin{array}{c} {\text{PAC}}_{\text{MVL}}=∥\frac{\bar{z}}{\text{max}(A(n))}∥, \end{array}$$ (2)

where $\bar{z}$ is the mean vector from the polar form in Eq (1) and normalized by the maximum amplitude in *A*(*n*). The mean vector is illustrated as the red line on the polar plot shown in Fig 1B. The MVL increases in magnitude as the polar form deforms asymmetrically, but approaches zero as it gains symmetry. This symmetry sensitivity is the main disadvantage of MVL as it cannot detect PAC if it is multi-modal and the modes are diametrically opposed in phases [8–10, 20, 21].

Phase-locking value (PLV) is defined as ∥mean(exp(*jϕ*_*p*_ − *jϕ*_*a*_))∥, where *ϕ*_*p*_ and *ϕ*_*a*_ are the phases of low-frequency signal and *A*(*n*), respectively [9, 10]. It can be observed that PLV is also a polar form representation with unit length radius, and it compares the synchronization of phases between two signals by quantifying the ratio between the two unit circles. PLV is 1 when the phases are locked, but nearly 0 when they are desynchronized. PLV is poor at differentiating small variations of PAC magnitude, but can detect the presence of PAC reliably [9, 10].

PAC can also be calculated from the phase-amplitude distribution (PAD) illustrated in Fig 1C. In PAD, A(n) is segmented according to *ϕ*(*n*) using *J* phase bins ($J\in Z_{+}$). Let PAD in bin *j* be defined as

$$\begin{array}{c} {\mathrm{PAD}}_{j}=\frac{{\bar{A}}_{j}}{\sum_{k=1}^{J}{\bar{A}}_{k}}, \end{array}$$ (3)

where ${\bar{A}}_{j}$ is the mean of *A*(*n*) segments that are in bin *j*. Note that the normalization applied in PAD_*j*_ characterizes PAD as a discrete probability density function (pdf). Absence of PAC in the raw signal results in a uniform distribution and the PAD diverges away from a uniform distribution as PAC increases.

Height ratio (HR) is the simplest technique used to quantify PAC from PAD. The HR is defined as

$$\begin{array}{c} {\text{PAC}}_{\text{HR}}=\frac{\text{max}(\mathrm{PAD})-\text{min}(\mathrm{PAD})}{\text{max}(\mathrm{PAD})}=1-\frac{\text{min}(\mathrm{PAD})}{\text{max}(\mathrm{PAD})}, \end{array}$$ (4)

However, because it only relies on the amplitude information of two phase bins (corresponding to the minimum and the maximum amplitudes in the distribution), it cannot differentiate between multi-modal or uni-modal PAC, it also cannot differentiate the modal width of PAC [9].

Modulation index (MI) quantifies PAC from PAD as

$$\begin{array}{c} {\text{PAC}}_{\text{MI}}=\frac{{\text{S}}_{KL}\left(\mathrm{PAD}\right)}{\ln\left(J\right)}, \end{array}$$ (5)

where

$$\begin{array}{c} {\text{S}}_{KL}\left(P\right)=\sum_{j=1}^{J}{\mathrm{PAD}}_{j}\ln\left(J{\mathrm{PAD}}_{j}\right), \end{array}$$ (6)

is the Kullback-Liebler (KL) divergence of PAD from a uniform distribution. This method is rigorously compared to other PAC quantifying techniques in [9]. Unlike HR, MI considers the shape of the PAD distribution for quantification. MI addresses the disadvantages of both HR and MVL [9].

Another quantification of PAC is the coefficients of correlation between amplitude and phase [9, 10]. Direct correlation with phase is sensitive to “null phases”, e.g., *ϕ*(*n*) = *k*/4, *k* = 1, 3, 5,…, so *A*(*n*) should be modelled with multiple regression of sin *ϕ* + cos *ϕ* [9, 10]. This technique assumes *A*(*n*) modulates sinusoidally rather than as periodic bursts of impulses. It was shown that this approach cannot reliably track changes in PAC.

Finally, the most recently introduced method for quantifying PAC is called the driven autoregressive (DAR) method [23]. This method uses a modified multivariate AR technique to model the power spectral density (PSD) of the high-frequency signal with the given low-frequency signal. PAC is quantified by measuring the level of fluctuations in the PSD using the KL divergence over a range of given phases. This method introduces additional complexity due to the necessity of keeping the AR filter stable at all times, since the AR filter coefficients are time-varying quantities. The key advantage of this method is that a probabilistic model is generated and used for PAC estimation, and thus a robust quantification can be made with a shorter signal segment.

#### Comodulogram

The described PAC quantification methods provide the magnitude for only a single pair of frequency bands. A phase-amplitude comodulogram can be used to visualize PAC values of multiple frequency pairs simultaneously. The comodulogram supports rapid visual identification of the frequency pair that exhibits the strongest coupling. The heat map in Fig 1D is a comodulogram using MI for PAC quantification. We generated comodulograms with *M* frequencies in the lower band and *N* frequencies in the higher band, yielding *M* × *N* frequency pairs. The original signal must be filtered *M* + *N* times to generate the analytical signals used to calculate all PAC values in the comodulogram. Although the comodulogram is computationally demanding, it is necessary when there are no a priori assumptions about the frequency bands of the phase-giving and amplitude envelope signal components.

### PAC innovations

Our PAC algorithms are packaged into a module for the Python programming language that we have named MSPACMan (Multirate Sub-banded Phase-Amplitude Coupling for Microelectrode Acquisitions with Noise), which is available at https://github.com/SachsLab/mspacman. There are currently other well-developed Python modules available for calculating PAC (i.e., pacpy and pactools). pacpy is available online at https://github.com/voytekresearch/pacpy, and pactools [21] is available at https://github.com/pactools/pactools. Both pacpy and pactools are not designed for real time online analysis and are not appropriate for our purposes, however we used them as a baseline comparison for our module. MSPACMan implements several optimizations during filtering and PAC quantification that make it suitable for online processing. MSPACMan performs filtering in the frequency domain, which enables the following optimizations:

- Fast Fourier Transform or FFT is performed only once per data segment;
- For each filter, a single element-wise multiplication of the frequency-domain signal is performed instead of a convolution of the time-domain signal;
- The desired output is the analytic signal which only requires positive frequency components, therefore only half of the data need to be multiplied by the filter’s frequency response;
- Filtering is achieved via vectorized broadcast multiplication in Numpy and parallelized processing across multiple channels or multiple frequency bands in a filter bank;
- Views of Numpy array are used to avoid copying of data.

The following additional optimizations are made for the PAC quantification step:

- The input data (low-frequency and high-frequency signal) are vectorized to enable broadcasting in Numpy and Scipy built-in functions;
- Parallel processing across multiple channels or multiple frequency bands are also enabled;
- Processing of compressed data (uneven decimation of low-frequency and high-frequency signal) are enabled to reduce the number of samples to process.

Further, MSPACMan separates its initialization from its signal processing. As long as the data segments to be processed have a consistent number of samples, calculating filter coefficients and allocating shared memory for parallel processes only need to be performed once during initialization.

#### Filter bank

**Fig 2:**
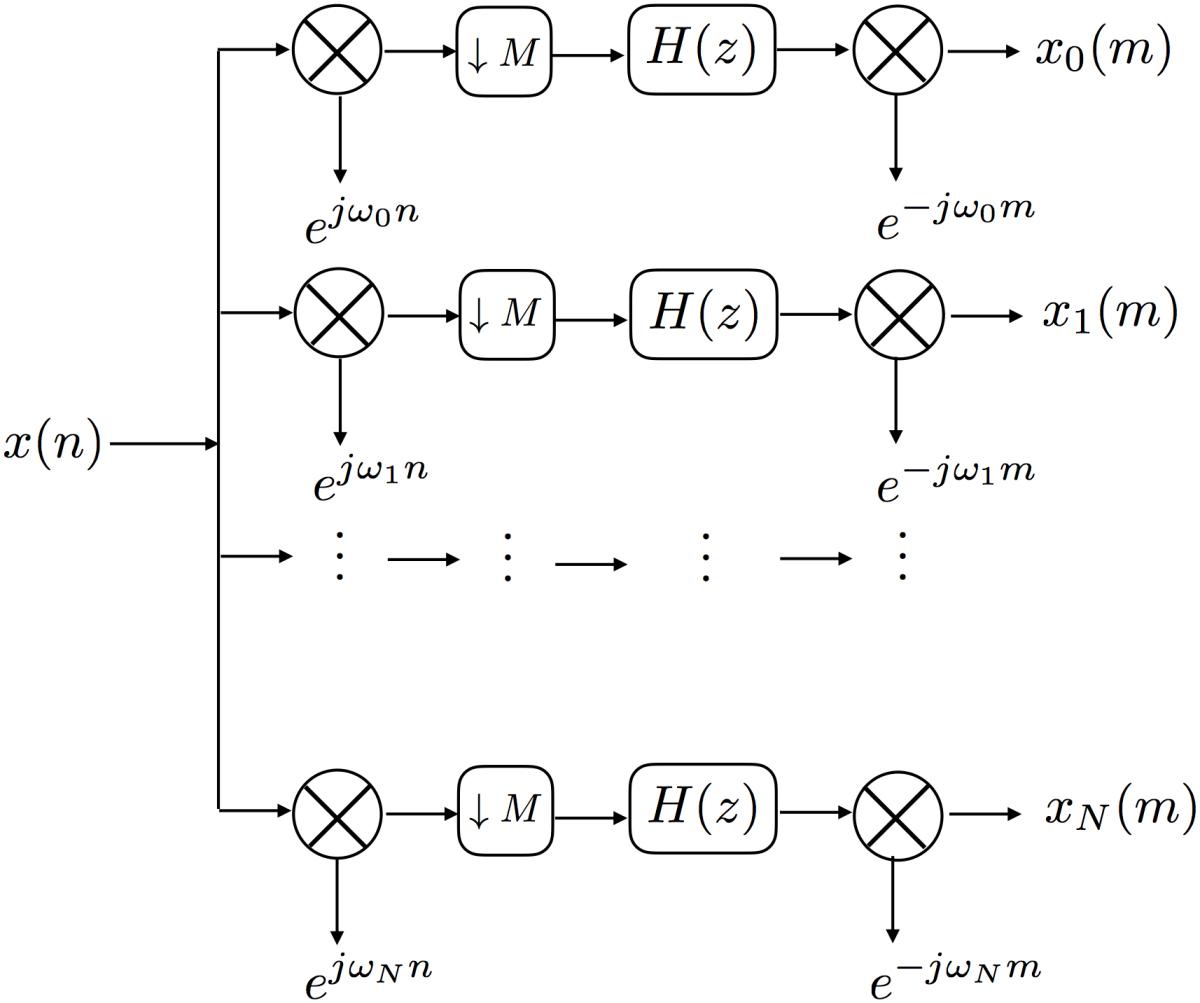
A block diagram representation of the filter bank. The block ⊗ → *e*^*jω*_*N*_*n*^ denotes that the signal’s frequency response has been shifted left such that *ω*_*N*_ is now at 0. The block ↓ *M* denotes the decimation in frequency with the factor *M*. The block *H*(*z*) denote the prototype filter. The block ⊗ → *e*^−*jω*_*N*_*n*^ denotes that the signal’s frequency response has been shifted from 0 back to *ω*_*N*_.

**Fig 3:**
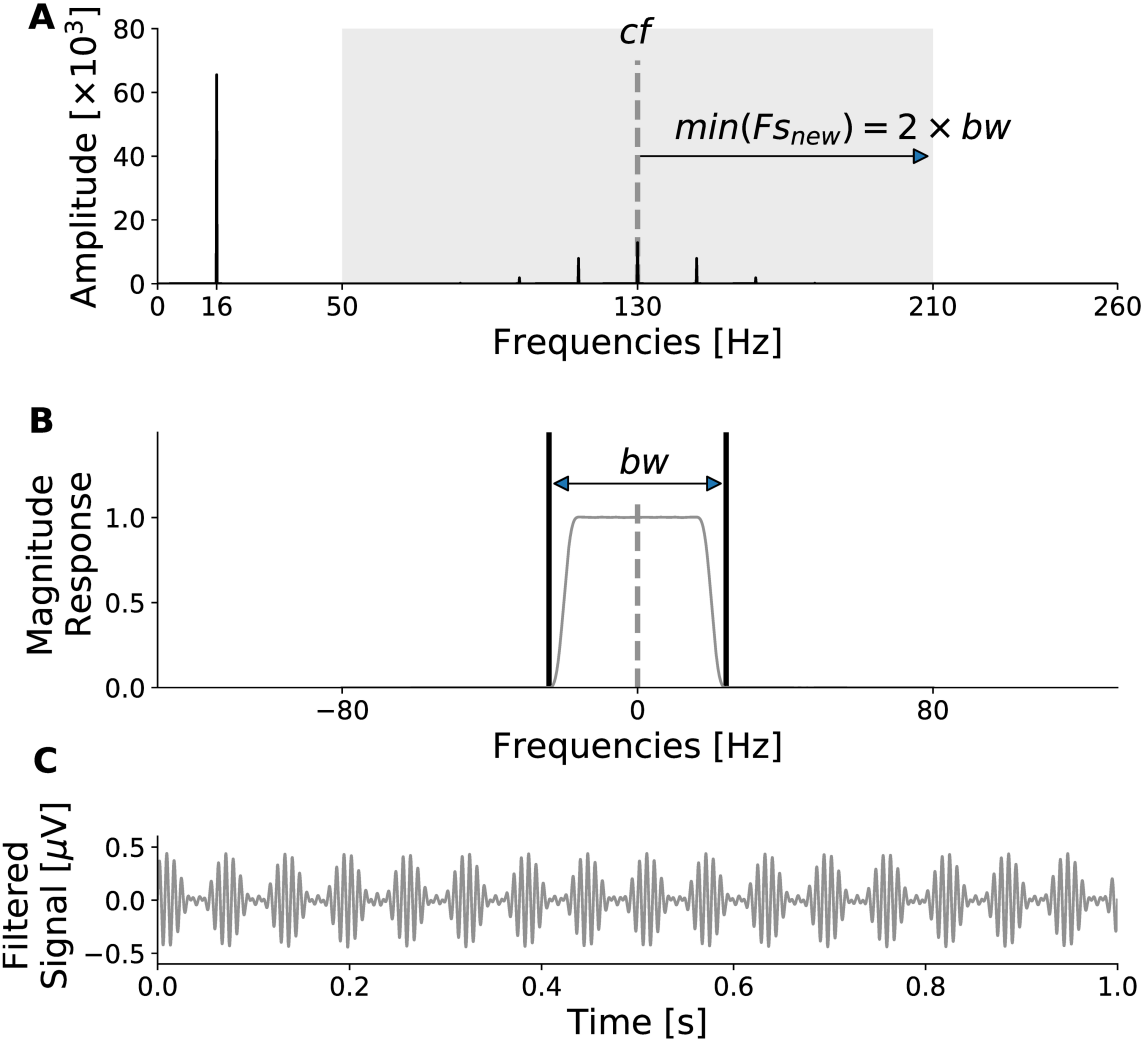
Illustration of the filtering process within each filter of the filter bank. A: The Fourier transform of the signal shown in Fig 1. The shaded patch indicates the frequency band of interest, centred on the filter’s centre frequency, *cf*. The edge of the patch is the Nyquist frequency after decimation, which is an integer fraction of the original sampling rate. B: The magnitude response of an 8000-tap FIR prototype filter. The patch falls completely under the length of the filter, where the frequency length under the passband is the bandwidth (*bw*). The Nyquist rate indicated must ideally be twice the bandwidth. Once the signal is shaped by the frequency response of the filter, it is shifted back to *cf* before applying inverse discrete Fourier Transform (IDFT). C: The filtered signal obtained after IDFT.

The input signal must be filtered independently *N* times, where *N* is at least 2 for a single phase-frequency amplitude-frequency pair, and possibly much larger for the calculation of the comodulogram. A filter bank is an array of bandpass filters that separates the input signal into *N* sub-banded signals. Efficient filtering from each filter is desirable for quick processing through the filter bank. Fig 2 shows a block diagram representation of an efficient parallel uniform modulated filter bank (UMFB) as a single-input *N*-output network. Fig 3 shows a filtered result of the signal shown in Fig 1 using the filter bank illustrated in Fig 2.

The UMFB can be realized as shown in Fig 2, where the frequency components of *x*(*n*) are shifted in frequency to the lowpass band, decimated in frequency (decimation factor *M* in Fig 2) by windowing 4 times the bandwidths about 0 Hz, multiplied by *H*(*z*), the frequencies are shifted back with the new sampling rate, and then the frequency components are transformed back to time domain signal, *x*_*i*_(*m*).

Frequency domain filtering is useful because it is faster (element-wise multiplication instead of convolution) when the input signal has many samples. Frequency domain filtering is especially useful in PAC calculation because it shares common steps with the calculation of the analytical signal required for PAC quantification. The Hilbert transform—a common method to obtain the time-domain analytical signal for PAC quantification—comprises frequency transformation, windowing, and inverse transformation. Filter coefficient multiplication can be built into the windowing step, turning the Hilbert transform into a filtering Hilbert transform. Further, filter coefficient multiplication can be performed on the frequency-domain signal independently for each filter, thus the FFT need only be applied once to the input signal, instead of to each output of the filter bank.

There are several conditions to be considered to properly design the filter bank for computing PAC. For simplicity, let us denote *H*(*z*) and *G*(*z*) as the prototype filters for obtaining the phase-giving and amplitude enveloping signal, respectively. It is desirable that the phase characteristics of the filters are as linear as possible because the phases of the different banded signals are being analyzed directly. As the signals are filtered in segments, it is possible to use non-causal zero-phase filtering (or forward-backward filtering) on each segment. The bandwidths for *G*(*z*) should be wide enough to capture all components including the side bands as shown in Fig 3. The duration of the segments and the amount of overlap depend on the application (online or offline) and desired frequency resolution.

In the design of the filter bank, each prototype filter (*H*(*z*) or *G*(*z*)) can be specified with any filter order, bandwidth, or filter type (FIR or IIR). A fifth order IIR Butterworth filter was used as the prototype filters in the computation of the comodulogram for offline analysis because it is maximally flat in the passband.

In realtime processing, the number of available samples increases over time; the signal must be segmented into constant length blocks for FFT filtering to reuse the previously initialized filter coefficients. The overlap-add method is an efficient way to perform FFT filtering of a long input signal with an FIR filter [22]. The overlap-add method can be used in theory on a zero-phase IIR filter of segments of signal that are zero-padded on both sides. The zero-padded lengths must be greater or equal to the length of the IIR filter’s impulse response (infinite length, which is practically impossible). In practice, the infinite length can be truncated at the point where the response drops below the desired noise floor, then the errors at the overlapping segments of the overlap-add method is related to the IIR length beyond the truncated segments. To reduce any further complexity, the prototype filter we used is now changed to an 8000-tapped FIR filter with linear phase characteristic.

#### Multirate phase-amplitude coupling

To process a signal quickly, it is common to compress signals (decimation) and process fewer data points. The phase-giving signal operates at a narrow frequency band, whereas the amplitude-enveloping signal requires a wide frequency band due to the need for capturing modulations in the amplitude. As a consequence, the phase-giving signal can be sampled at a lower rate than the amplitude-enveloping signal, resulting in uneven lengths of signals. Currently available methods for estimating PAC magnitude, however, require the phase-giving and the amplitude-enveloping signals to have the same length. We circumvented this limitation by setting the amplitude-enveloping signal sampling rate to an integer multiple of the phase-giving signal rate. We then upsampled the phase values to match the rate of amplitude values.

### Test environment

We developed and tested MSPACMan using generated signals and microelectrode recordings (MERs) obtained during DBS surgery.

#### Generated signals

The generated signal, represented in a sequence, is composed of two elementary sinusoidal sequences

$$\begin{array}{c} x\left(n\right)=x_{p}\left(n\right)+x_{a}\left(n\right), \end{array}$$ (7)

where

$$\begin{array}{ccc} x_{p}\left(n\right) & = & \sin\omega_{p}n, \end{array}$$ (8)

$$\begin{array}{ccc} x_{a}\left(n\right) & = & A_{a}\left(n\right)\sin\omega_{a}n, \end{array}$$ (9)

$$\begin{array}{ccc} A_{a}\left(n\right) & = & \frac{1}{4}\left(P\sin\left(\omega_{am}n+\pi \right)+2-P\right), \end{array}$$ (10)

$$\begin{array}{ccc} \omega_{i} & = & 2\pi \frac{F_{i}}{F_{s}}, \end{array}$$ (11)

*F*_*p*_, *F*_*a*_, *F*_*am*_ are frequencies characterizing periodic sequences, *F*_*s*_ is the sampling rate, and *P* is the magnitude of PAC ranging between 0 and 1. In this study, the parameters are set as *F*_*p*_ = *F*_*am*_ = 16 Hz (in the *β* band), *F*_*a*_ = 130 Hz (in the *γ*_2_ band), *F*_*s*_ = 16, 384 Hz, and *P* remained as a controllable variable depending on simulation scenarios.

Eq (9) assumes that *A*_*a*_(*n*) modulates sinusoidally, but it could instead be modulated as periodic bursts. This can be modelled by convolving an impulse train (periodic impulse functions, *δ*(*n*)) to a Gaussian function

$$\begin{array}{c} f\left(n\right)=\frac{1}{\sigma \sqrt{2\pi }}\exp\frac{-{\left(n-\mu \right)}^{2}}{2\sigma^{2}}, \end{array}$$ (12)

where *μ* and *σ* are the mean and standard deviation of the Gaussian distribution, respectively, which shape the Gaussian function of each burst. The equation for the signal generator, Eq (9) is modified as

$$\begin{array}{c} A_{a}\left(n\right)=f\left(n\right)*\sum_{p\in Z}\delta \left(n-pT_{am}\right), \end{array}$$ (13)

and

$$\begin{array}{c} T_{am}=F_{am}^{-1}. \end{array}$$ (14)

In practice, some common noise sources are observed in recorded biological signals. For example, repeated measurements of the same signal varies based on statistical variances, this is referred to as measurement noise. Measurement noise can be replicated from values drawn from a zero mean white Gaussian noise of a random process, and thus have the property of constant intensities across all frequencies.

The measurement of a signal emitted from a biological source is often corrupted by the echoes of neighbourhood sources. Biological noise, or pink noise (1/*f* noise), is also modelled from a random process with the frequency domain property where the magnitude is inversely proportional to the corresponding frequencies. These noise models are added to the signal in Eq (7) to approximate a recorded signal.

#### Recorded signals

The MER signals were recorded using the AlphaOmega Microguide Pro (Alpha Omega, Inc, Nazareth, Israel) from microelectrodes inserted into the putative STN during the neurosurgical procedure to implant DBS electrodes. The signals were amplified 10,000-fold and digitized at 24 kHz with 12-bit resolution. In this study we use a single 3-channel trajectory from one hemisphere from one patient. Use of anonymized intraoperative patient data for this study has been approved by the Ottawa Health Science Network Research Ethics Board.

#### Sensitivity analysis

MSPACMan, pactools, and pacpy were compared on their ability to compute a MI comodulogram. We quantify the performance from the determination of PAC-containing frequency pairs from the comodulograms, from the ability to track changes in PAC, and finally the processing time to calculate each comodulogram.

PAC-containing frequency pairs were determined using a simple threshold-based blob detection algorithm on the comodulograms. The blob detection algorithm identifies pixel groups with MI above the threshold. The blob centre denotes the main contributing frequency pairs of PAC in the test signal. This detection algorithm is only useful for simulated signals because it is overly sensitive to noise.

Signals were simulated four times at each of 10 levels of specified PAC (from 0.0 to 1.0) and 10 levels of noise, specified by signal to noise ratio (SNR) of oscillatory amplitude to standard deviation of the noise, (from SNR 10.0 to SNR 1.0). For each algorithm, the comodulogram was calculated and the average MI value in the detected blob was averaged across the four repetitions and used as the PAC output value. PAC output values were normalized within-algorithm to the range of PAC values calculated at SNR 10.0.

We also quantified the processing time of the comodulograms. Unlike MSPACMan which exposes distinct initialization, filtering, and PAC quantification, pacpy and pactools perform the entire process from initialization to PAC quantification in one top-level function. Therefore, to compare processing time across the different modules, the measured processing time includes both the filter bank and PAC quantification. Each process was performed 40 times, and the mean and the standard deviation of the computation time were reported.

The machine used to test computation times is a 2012 MacBook Pro with a 2.5 GHz dual-core Intel Core i5 processor. The PAC algorithms and analysis tools were written in Python 3 using packages for numerical and scientific processing (Numpy and Scipy) optimized with the Intel Math Kernel Library.

### Results

#### Comparing different Python modules for PAC

**Fig 4:**
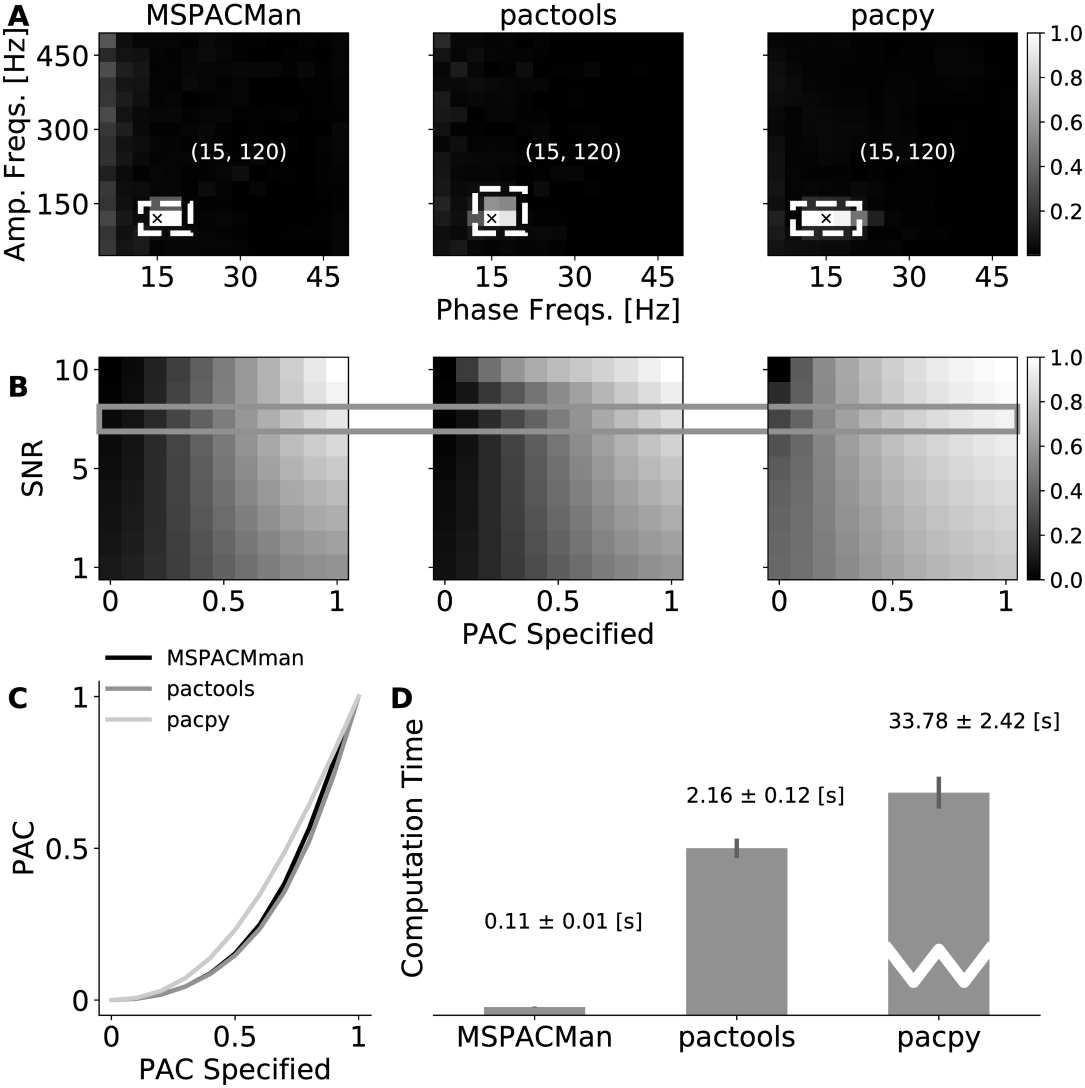
Comparison of MSPACMan with other existing PAC packages. A: 15 × 15 comodulograms with the suprathreshold PAC blobs identified. B: Normalized PAC magnitudes (MI) at the frequency pairs identified in A, calculated for multiple levels of specified PAC and noise-level in the simulated signal. C: Overlay of normalized PAC magnitudes for the three different methods at varying levels of specified PAC and a constant SNR of 6:1. D: The processing time for computing each comodulogram.

Two other Python modules for computing PAC were identified: pacpy and pactools. We compared the MI comodulograms computed using MSPACMan, pactools, and pacpy, shown in Fig 4A. For each method, the automated blob detection algorithm detected the centre of peak PAC close to the specified frequency pair for the test signal. The specified PAC frequency pair was (16, 130) Hz and all modules detected peak PAC near that frequency pair (MSPACMan (15, 120) Hz; pactools (15, 120) Hz; pacpy (15, 120) Hz). For each PAC module, the average MI value from the blob was calculated at multiple levels of specified PAC and noise (Fig 4B). Detected PAC magnitude at the identified frequency pair(s) was positively related to the specified PAC magnitude in the simulated signal and negatively related to the amount of noise in the signal. The relationship between input PAC and calculated PAC at a constant signal-to-noise ratio (SNR) of 3.0 is plotted for all three methods in Fig 4C. Calculated PAC values were similar across methods, except pacpy yielded greater PAC at middle ranges of specified PAC. Fig 4D shows the time required for each Python module to compute a 15 × 15 comodulogram. MSPACMan required 0.1 s, pactools required 2.2 s, and pacpy required 33.8 s.

#### Realtime applications of PAC calculation

**Fig 5:**
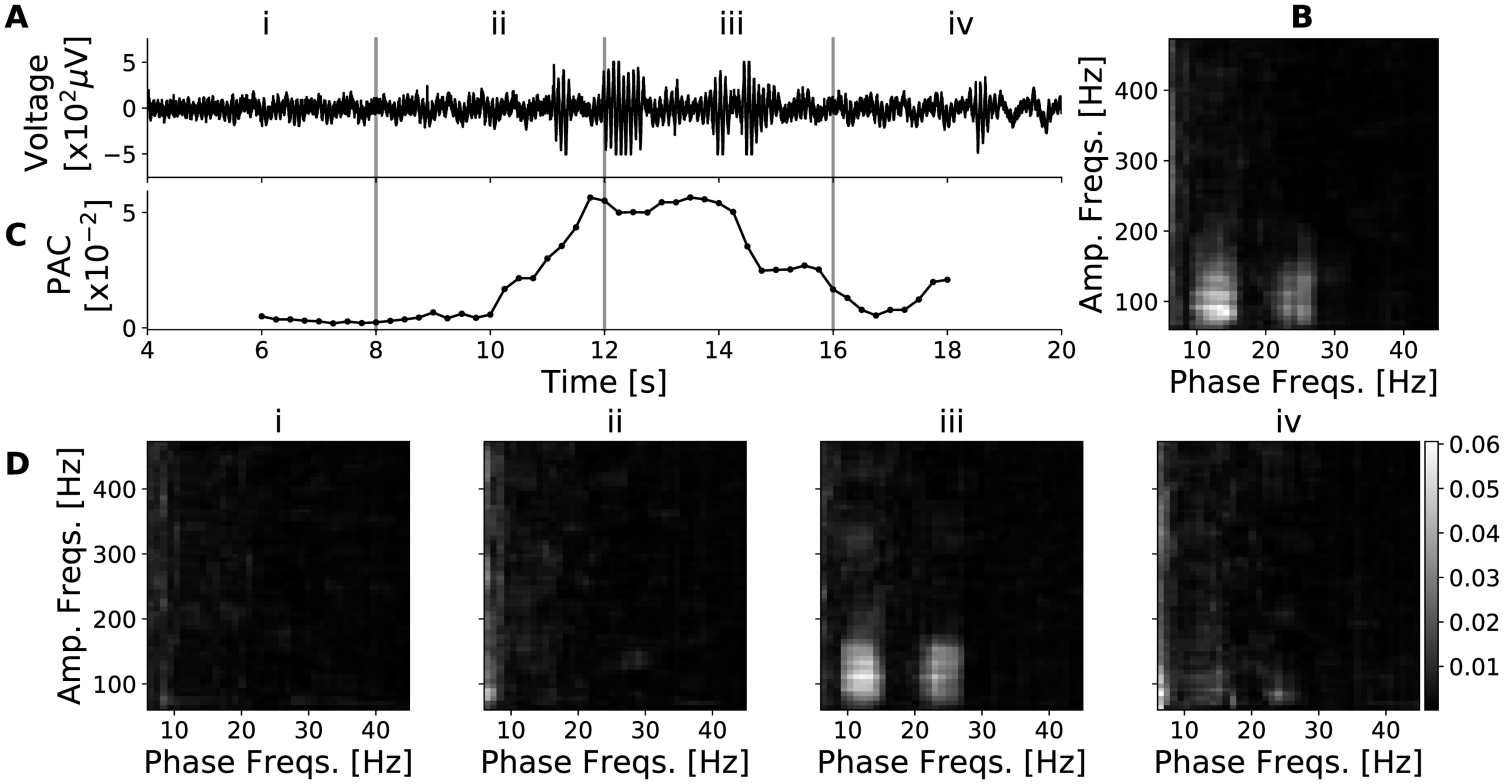
Estimates of PAC in window segments of a signal recorded during an STN-DBS surgery. A: 16 seconds duration of a raw voltage signal. B: The comodulogram of the full length of A. C: The *β* − *γ*_2_ PAC of A calculated in 4-second windows with a step size of 250 ms (i.e., 95% overlap). D: The comodulograms of A in non-overlapping segments of 4-second windows indicated.

We calculated PAC magnitude of a signal recorded during an STN-DBS surgery (Fig 5). In the comodulogram calculated from the entire 16-second duration of the signal (Fig 5B), PAC magnitude peaks with the phase-giving frequencies of 6-50 Hz and amplitude-envelope frequencies of 60-500 Hz. PAC magnitude for this frequency pair calculated from shorter signal segments varies significantly across time (Fig 5C). Selected comodulograms from short segments (Fig 5D) demonstrate that PAC magnitude at the frequency-pairs of interest was transient and that PAC magnitude at other frequencies was not observed.

## Discussion

We developed MSPACMan, a signal analysis module for the Python programming language that can calculate phase amplitude coupling quickly and robustly and is suitable for realtime analysis of neurophysiological signals. MSPACMan accelerates PAC calculation by several modifications to traditional PAC algorithms including embedding of the Hilbert transform in a frequency domain filtering step, signal decimation, and parallelization. We compared MSPACMan to other available PAC analysis algorithms and found that our approach is 20-300 times faster and yields results that are qualitatively similar or better.

MSPACMan allows the user to specify the PAC quantification method. In this study, we used exclusively the MI as the PAC quantification method. MI is commonly used in the neuroscientific literature as the primary PAC measure due to its tolerance to noise, sensitivity to multimodality in PAD, sensitivity to the modulation widths of the amplitude-modulation, and its ability to reliably track the changes in PAC intensities [9].

When the modulating and modulated frequencies are not known in advance, it may be necessary to calculate a comodulogram of PAC values for all frequency combinations. Comodulograms obtained from MSPACMan were similar to those obtained from pactools, and both identified a smaller set of PAC-containing frequency pairs than pacpy, though all three packages identified the same peak frequency combination closest to the specified combination. The precision of the identified peak frequency combination can be made more precise by increasing the frequency resolutions of the comodulogram.

For all three tested methods, the calculated PAC value had a monotonically increasing relationship with the specified PAC magnitude at low levels of noise. In general, as the amplitude of the noise in the generated signal increased, the calculated PAC magnitude decreased. However, for pacpy, at low levels of specified input PAC, calculated PAC magnitude increased as noise increased, suggesting that noise might be contributing to spurious PAC detection.

We tested MSPACMan for its ability to calculate PAC from neurophysiological data recorded from MERs in the STN of patients undergoing DBS surgery. While PAC was evident when analyzing a long signal segment (16 s), when examined more precisely it was only present in one 4-second segment within the longer segment (Fig 5). MSPACMan can measure PAC from 4-second window segments and it can use small time steps between updates of the output PAC value because it is fast (Fig 5B). We further demonstrated that MSPACMan is sensitive enough to identify the lack of PAC in the initial 4-second window segment, to its emergence in subsequent segments, and its return to baseline shortly thereafter (Fig 5C and 5D).

An important consideration for a robust realtime signal processing tool is its ability to handle signal artefacts with minimal impact on its output. PAC analyses are commonly applied to electrocorticography (ECoG) signals, in which the electrodes are located on the surface of the brain. As such a variety of environmental or experimental artefacts are commonly encountered. However, an intracortical signal like the MER has a significantly higher SNR, and thus signal artefacts are less common. The two that are most commonly seen in our data are short duration bursts induced by mechanical disturbances (e.g. patient tremor that shakes the lead wire) and signal clipping due to the recorded signal amplitudes exceeding the hardware tolerance.

We tested MSPACMan for its ability to calculate PAC from artefact-contaminated neurophysiological data by artificially generating artefacts and comparing performance between the original data with that artefact-infused data. The simulated artefacts mimicked the quantity and quality of the occasional signal clipping that occurs during neurosurgical microelectrode recording. Although small differences are observed, they are insufficient to justify preprocessing with tools to detect and replace such artefacts. We also performed the comparisons with pactools and pacpy (see the results at S1 and S2 Figs) with similar results.

Two important applications of realtime PAC calculation enabled by MSPACMan are for the treatment of PD either as a trigger for closed loop DBS [16, 17] or as a neurofeedback target to induce and guide adaptive plasticity using brain-computer interfaces [15]. It was demonstrated that PAC quantified in putative human STN can be transient, emerging in the signal for only a few seconds (Fig 5). This is important because both of these applications that requires temporal precision in PAC onset will require PAC quantification from small window of data and rapid updates. Although this property was commonly observed in the data we examined, its occurrence probability study is outside the scope of this manuscript.

## Conclusion

We developed a Python module, MSPACMan, for realtime calculation of phase-amplitude coupling. The algorithm details are provided and its performance is compared to already available Python modules for PAC. We tested MSPACMan with generated and clinical signals. We demonstrated that MSPACMan can calculate PAC and the comodulogram much faster than currently available solutions, and that it reliably detects and quantifies PAC of a given signal. The fast and robust calculation of PAC magnitude provided by our module may have clinical applications and will enable future studies into the clinical relevance of PAC modulation on short time scales. The algorithms developed herein may be of additional benefit in fields such as computer vision, finance, and geophysics that require fast processing of spectral and synchronization features of multichannel time series data.

## Supporting information

S1 Fig Comparison of MSPACMan with pactools and pacpy on the effect of clipping. A) A 16-second duration of the original raw voltage signal (blue), and a artificially clipped version of the same signal (orange). B) The *β*-*γ*_2_ PAC of both signals in A) calculated in 4-second windows with a step size of 250 ms (i.e., 95% overlap). C) The comodulogram of the full lengths signals in A).
(TIF)

S2 Fig Comparison of MSPACMan with pactools and pacpy on the effect of signal artefacts. A) A 16-second duration of the original raw voltage signal (blue), and a artificially generated spiking artefacts added the same signal (orange) at 6 s and 12 s with 0.5 s of bursts. B) The *β*-*γ*_2_ PAC of both signals in A) calculated in 4-second windows with a step size of 250 ms (i.e., 95% overlap). C) The comodulogram of the full lengths signals in A).
(TIF)

S1 Appendix Descriptions of simulating and the PAC processing of simulated artefacts signals. (PDF)

S1 Dataset The data files required to replicate the results of this report. There are two .mat files compressed into S1_Dataset.zip.
(ZIP)
