# Supplementary figures and images for: Realtime phase-amplitude coupling analysis of micro electrode recorded brain signals
Source: PLoS One. 2018 Sep 28;13(9):e0204260. doi: 10.1371/journal.pone.0204260 (PMC6161890; doi:10.1371/journal.pone.0204260)

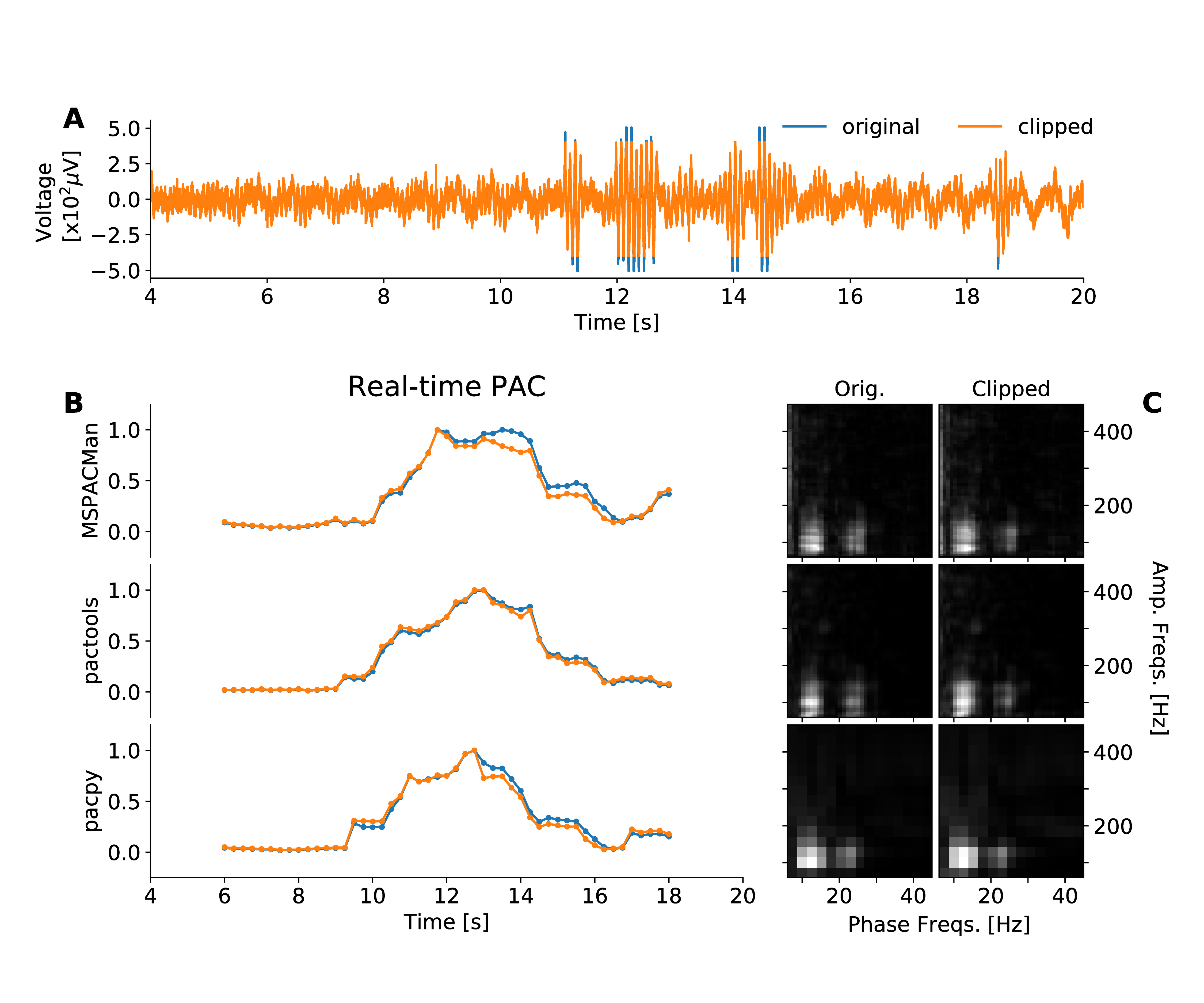

Supplement: S1 Fig — A) A 16-second duration of the original raw voltage signal (blue), and a artificially clipped version of the same signal (orange). B) The β-γ2 PAC of both signals in A) calculated in 4-second windows with a step size of 250 ms (i.e., 95% overlap). C) The comodulogram of the full lengths signals in A). (TIF) [file pone.0204260.s001.tif]

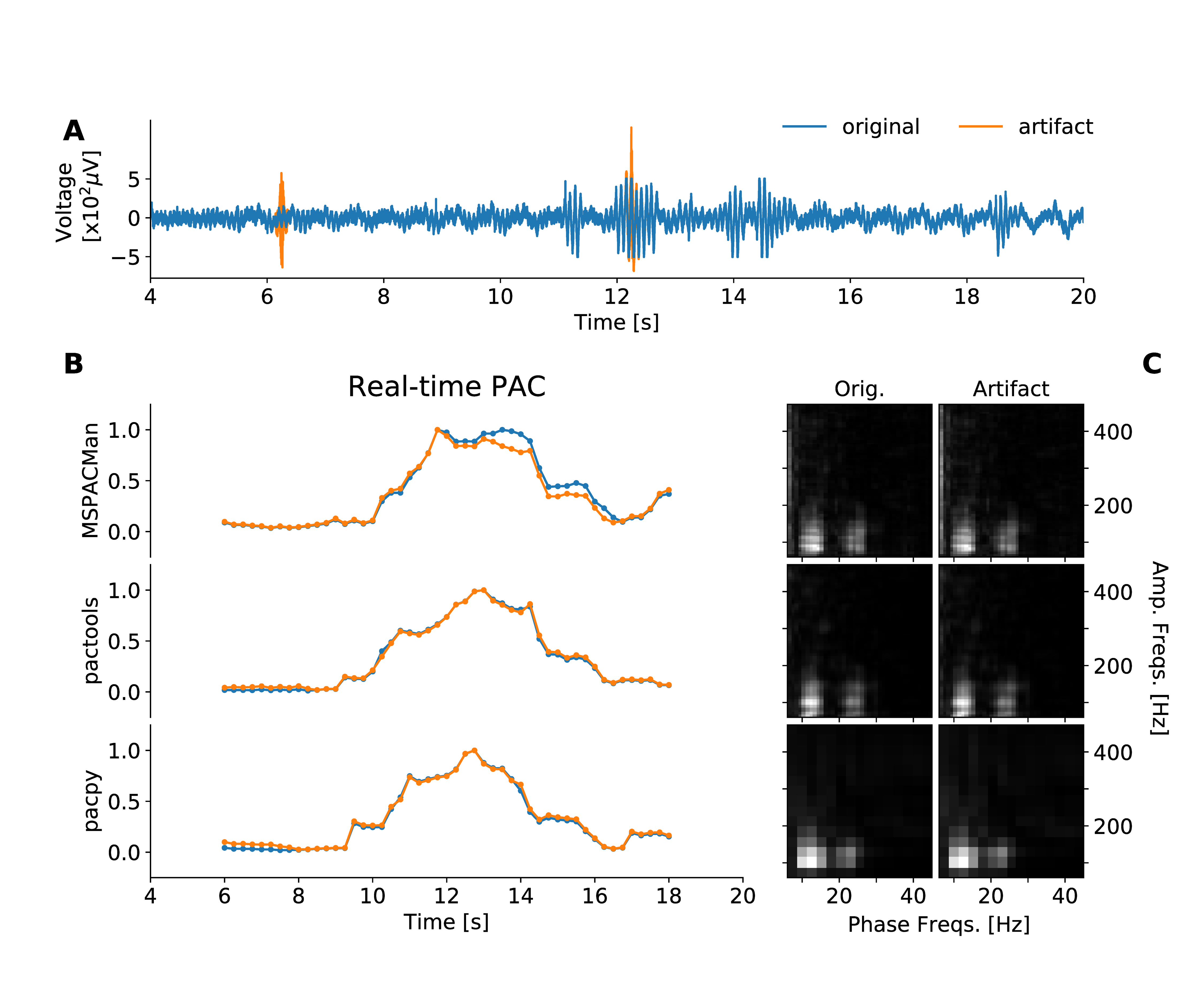

Supplement: S2 Fig — A) A 16-second duration of the original raw voltage signal (blue), and a artificially generated spiking artefacts added the same signal (orange) at 6 s and 12 s with 0.5 s of bursts. B) The β-γ2 PAC of both signals in A) calculated in 4-second windows with a step size of 250 ms (i.e., 95% overlap). C) The comodulogram of the full lengths signals in A). (TIF) [file pone.0204260.s002.tif]
