# Supplementary material for: Realtime phase-amplitude coupling analysis of micro electrode recorded brain signals
Source: PLoS One. 2018 Sep 28;13(9):e0204260. doi: 10.1371/journal.pone.0204260 (PMC6161890; doi:10.1371/journal.pone.0204260)
Supplement: S1 Appendix — (PDF) [file pone.0204260.s003.pdf]

S1 Appendix

In this section we provided the results of the analysis that demonstrate the effect of signal artifacts to the PAC calculations performed with MSPACMan. Additionally, we performed the same analysis on the existing PAC tools used in other analyses in this study, pactools and pacpy. The results are summarized in S1 Fig and S2 Fig.

As mentioned in the manuscript, the two main artifacts encountered in our data are due to clipping and/or motion artifacts. We took the data processed in the manuscript, and clip the signal at ± 400 µV . We then process the signal using MSPACMan for the results of single frequency pair PAC, as well as the comodulogram. As shown in the results, there are not much differences in the results between both PAC forms.

The second form of artifact was simulated and added to the same data. The motion artifact was generated by taking a 0.5 second burst of 5 Hz sinusoid and multiply element-wise by a random generated data extracted from $\mathcal{N}(0, 1)$, the resulting signal is then shaped by a gaussian kernel of $\mathcal{N}(0, 0.05)$. This burst of signal is then added to the original signal at 6 seconds and at 12 seconds. As shown in the results, there are not much differences in the results between both PAC forms either.

Due to the lack of significant differences between the results with and without artifacts, the analyses regarding the topic of artifacts were removed from the main manuscript. However, the analyses are provided in `https://github.com/davidlu89/notes_mspacman`.
